# Heat generation of autologous bone harvesting drills: an in vitro study

**DOI:** 10.1038/s41598-026-35988-3

**Published:** 2026-01-13

**Authors:** Fanni Jáni, Nick Köhler, Edina Lempel, Péter Szabó, Stephan Christian Möhlhenrich, József Szalma

**Affiliations:** 1 Department of Oral and Maxillofacial Surgery, Medical School, University of Pécs, 1. Tüzér St., Pécs, H-7623 Hungary; 2 Department of Conservative Dentistry and Periodontology, Medical School, University of Pécs, 1. Tüzér St., Pécs, 7623 Hungary; 3 János Szentágothai Research Center, Ifjúság Street 12, Pécs, 7624 Hungary; 4 Department of Dentistry, Section of Orthodontics, Dentofacial Orthopedics and Craniofacial Biology, Radboud University Medical Centre, 309, P.O. Box 9101, Nijmegen, 6500 HB The Netherlands

**Keywords:** Autologous bone chip, Bone harvesting, Bone temperature, Drilling speed, Axial load, Drill wear, Engineering, Health care, Medical research

## Abstract

Bone collecting drills are utilized in implantology to harvest autologous bone chips. This study aimed to assess intraosseous and bone chip temperature changes during bone collecting procedures. Auto-Max (Megagen, Luton, UK) drills were evaluated at rotational speeds of 300, 600, 1200, and 2000 revolutions per minute (rpm), each combined with axial loads of 15 N, 20 N, and 25 N, using fresh pig ribs. Axial load along the perpendicular axis was maintained via a dedicated drilling tower. Bone chip temperatures were measured using an infrared non-contact thermometer, whereas donor bone temperatures were monitored with thermocouple sensors positioned 0.5 mm from the osteotomy periphery. Drill wear was assessed through scanning electron microscopy. The highest donor bone temperature observed was 10.1 ± 3.04 °C at 1200 rpm and 25 N. With a 20 N load, temperature increases ranged from 3.01 to 4.84 °C. At 25 N, temperatures at 300 rpm (1.84 ± 0.58 °C) and 600 rpm (6.70 ± 2.64 °C) consistently stayed below 10 °C. Bone chip temperature rises were all under 5 °C. The slowest drilling occurred at 15 N/600 rpm (6.49s), and the fastest at 2000 rpm (< 1.65s). Drill wear was moderate up to 10 uses, but significant after 30, with bone temperatures rising by 240% to an average of 10.22 ± 2.45 °C. With respect to chip temperatures, all evaluated drilling parameters produced maximum temperature elevations below 5 °C. Donor bone temperature increases remained consistently under the theoretical threshold of 10 °C when an axial load of 20 N was applied—regardless of rotational speed—or when drilling at 600 rpm, independent of axial load. It is recommended that drills be replaced prior to 30 uses.

## Introduction

Bone preparation is inherently a highly intricate mechanical process. Heat generation during drilling predominantly occurs through three mechanisms: (i) cutting and plastic deformation of the material by the cutting edge, (ii) friction generated between the drill body and the bone, and (iii) friction between the chip and the hole wall^1^. Furthermore, bone exhibits low thermal conductivity, which limits the dissipation of generated heat. Thermal damage to bone tissue, including thermal osteonecrosis, can occur if the temperature exceeds 47 °C for one minute; however, exposure to higher temperatures results in necrosis over significantly shorter durations^2,3^. In addition to thermal effects, high thrust force (axial load) can contribute to the development of microcracks^4^, and drilling forces can have a significant impact on the length of these cracks^5,6^. Thermal and mechanical trauma are known to substantially impact bone healing processes, potentially leading to disturbances^7–9^, as well as affecting implant osseointegration^10^.

The generation of heat during drilling is primarily determined by two key factors: the drilling parameters and the specifications of the drill itself^10–12^. Drilling parameters encompass variables such as rotational speed, feed rate, type of cooling (external, internal, mixed, or cooled irrigation), drilling depth, and whether gradual drilling is employed. Drill specifications are largely established by manufacturers and include attributes such as material composition, diameter, rake angle, clearance angle, cutting edge and flute design, point angle, and chisel edge configuration^10,13,14^. Furthermore, certain related parameters—such as bone density or the degree of drill wear—can exhibit significant variability and present challenges for precise quantification^9^.

The choice of instrumentation for bone harvesting is widely addressed in the literature^15–18^. Studies indicate that, during autogenous bone harvesting, manual instruments and piezosurgery produce more viable cells than rotary devices; in the referenced study, the rotary technique used 2.2 mm diameter round drills^18^. Additionally, Miron et al. reported that both the bone scraper and trephine (combined with bone milling) demonstrated higher cell viability and greater release of bone-forming factors (BMP-2, VEGF, NF-κB) compared to piezosurgery or bone drilling^16^.

Most studies investigating drills are conducted in vitro or ex vivo. This approach allows for standardization of all parameters except the specific drilling parameter being studied, which is challenging to achieve intraorally with a handheld instrument in living subjects. Additionally, these in vitro studies utilize various bone models, including those derived from humans, animals, or synthetic sources. A previous study comparing bone models found that fresh porcine bone samples showed similar properties to human bone, whereas bovine bone samples exhibited higher temperatures under the same drilling conditions^19^. Furthermore, drilling conducted on artificial polyurethane bone models resulted in the production of “non-adhesive, powder-like” particles, which contrasts with observations made during in vivo bone preparation procedures^9,19^.

During drilling, a major factor that reduces heat is the removal of prepared fragments as they exit the cavity. In contrast, bone collecting drills do not enable this heat reduction, since bone fragments accumulate within the inner space of the drill, possibly leading to increased heat during the drilling process. Additionally, irrigation serves as an effective method for reducing temperature; however, its use is not always mandated by manufacturers in the context of bone collecting drills, as this could increase the risk of losing the collected bone chips. While the temperature-raising effects of various dental and surgical drills—such as implant drills^14,20,21^, mini-implant predrills^22^, tungsten carbide round drills^9,13,19^, trephine drills^23^, and tooth sectioning drills^24^—have been studied, there is still limited evidence regarding bone collecting drills.

The aim of this study was to investigate the relationship between the two most important drilling parameters, namely rotational speed and axial load and the intraosseous heat generation in the donor bone and in the harvested bone chips during bone collecting preparations. A further aim was to observe the effect of drill wear on temperature increases.

## Materials and methods

### Experimental set-up

Cavities measuring 4 mm in depth were created in fresh pig rib segments using bone collecting drills (Auto-Max^®^, MEGAGEN Implants, Luton, United Kingdom) with a diameter of 3.5 mm. The study examined rotational speeds of 300, 600, 1200, and 2000 revolutions per minute (rpm) in combination with axial loads of 15 N, 20 N, and 25 N.

**Fig. 1:**
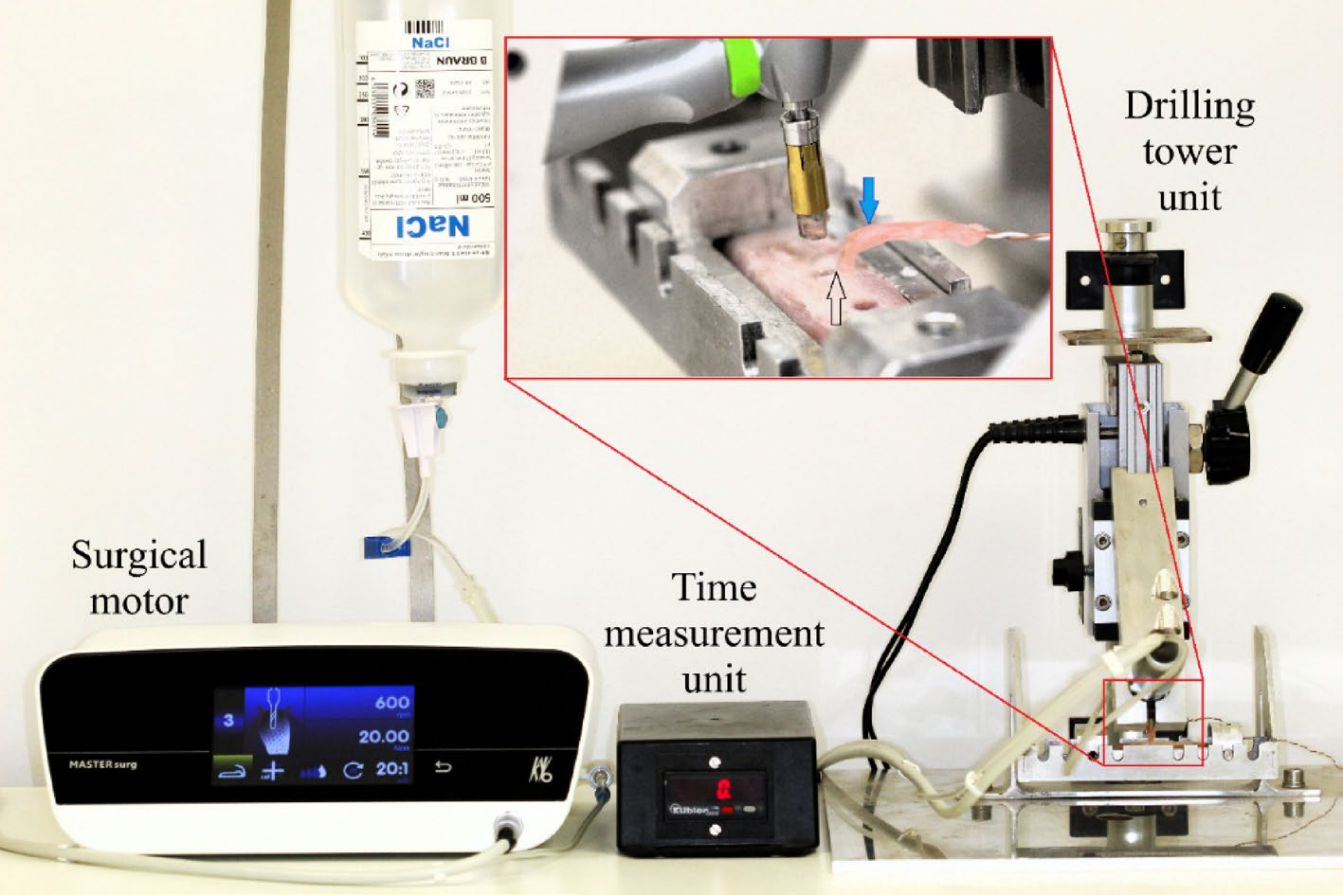
An experimental drilling tower apparatus was used to control the precise movement of the surgical contra-angle handpiece. In the magnified area, the thermocouple probe in wax isolation (blue arrow) is shown positioned close to the axial wall of the bone preparation (black arrow).

The drilling axis was oriented perpendicular to the bone surface, and the axial load applied during preparation was standardized using a custom-designed drilling tower^9,13,19^. A surgical 20:1 implant handpiece (WS-75 LG, W&H, Bürmoos, Austria) was securely mounted in the axial motion unit of the tower and operated by a surgical motor (Mastersurg Lux Wireless, KaVo GmbH, Biberach/Riss, Germany) for the duration of the experiment (Fig. 1). The drilling time was measured automatically, by a magnet-controlled electronic time counter (Kübler Codix, Villingen-Schwenningen, Germany).

Preparations were evaluated using rotational speeds of 300, 600, 1200, and 2000 rpm combined with axial loads of 10, 15, and 25 N. The specific conditions tested were: 300 rpm-25 N; 600 rpm-15 N, 20 N, 25 N; 1200 rpm-15 N, 20 N, 25 N; and 2000 rpm-15 N, 20 N, 25 N. Application of axial loads of 15 N and 20 N at a rotational speed of 300 rpm, the bone collecting drill was incapable of achieving cortical penetration in fresh porcine rib specimens.

Each group underwent twelve drilling procedures, resulting in a total of 120 bone chip collection sites (ten groups multiplied by twelve repeats/group) being prepared. All drills prepared only 12 cavities. Following the previously described drilling session, drills were used to prepare an additional eighteen cavities. Following a total of 30 cavity preparations, the temperature increase of the worn drills—still intact and not yet fractured—was assessed under conditions identical to those used for new drills, specifically at 20 N force and 600 rpm. Irrigation was set to ~ 90 ml/min during all preparations.

### Bone sample preparation

For the experiment, fresh porcine ribs were bought by the butcher. Animals were not sacrificed because of this experiment! Bone samples were from the same, 10-month-old male animal. Ribs were cut to 5 cm long sections and further selected according to comparable cortical thicknesses (2.2 ± 0.1 mm). Cortical thickness was measured on the cutting surfaces with a digital caliper. Each individual bone segment permitted four distinct preparations within the tower, with drilling parameters allocated randomly among the segments for each preparation. With this method, all the drilling groups investigated were tested in several (> 5) bone samples.

### Temperature measurement

**Fig. 2:**
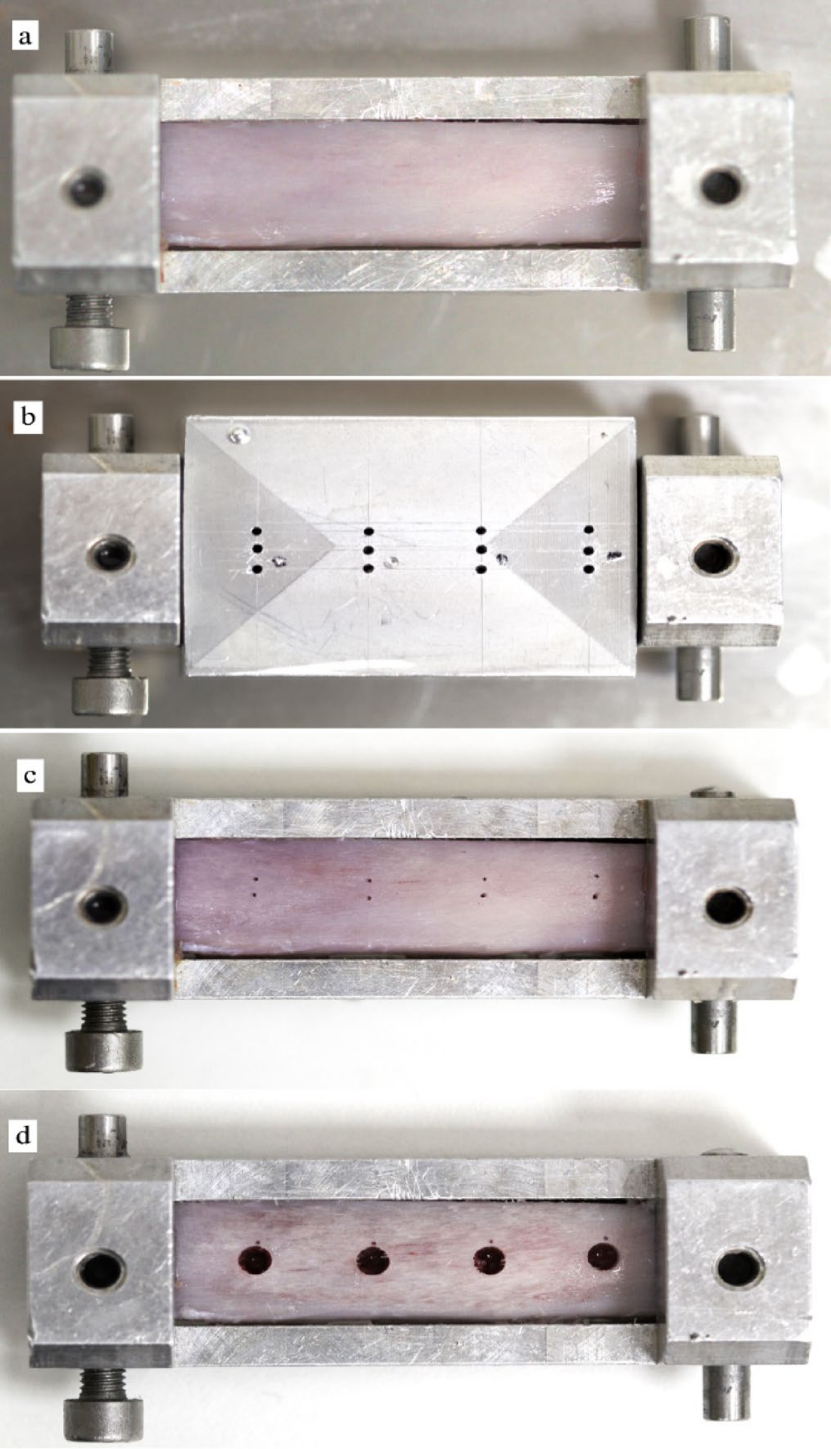
Porcine bone specimens were secured using a metal vice **(a)**, which was positioned at predetermined locations within the drilling tower to ensure consistent alignment between cavity preparations and thermal sensor placements. Thermal sensors were installed according to the positions specified by the metal guide fitted onto the vice **(b)**. The centers of bone harvesting cavities were marked, with sensor locations identified adjacent to each site prior to drilling **(c)**. Following completion of cavity preparation, a uniform distribution of thermosensor placements was evident **(d)**. Randomizing drilling positions in the specimen for different study groups could effectively compensate for minor differences in distances between cavities and sensors.

**Fig. 3:**
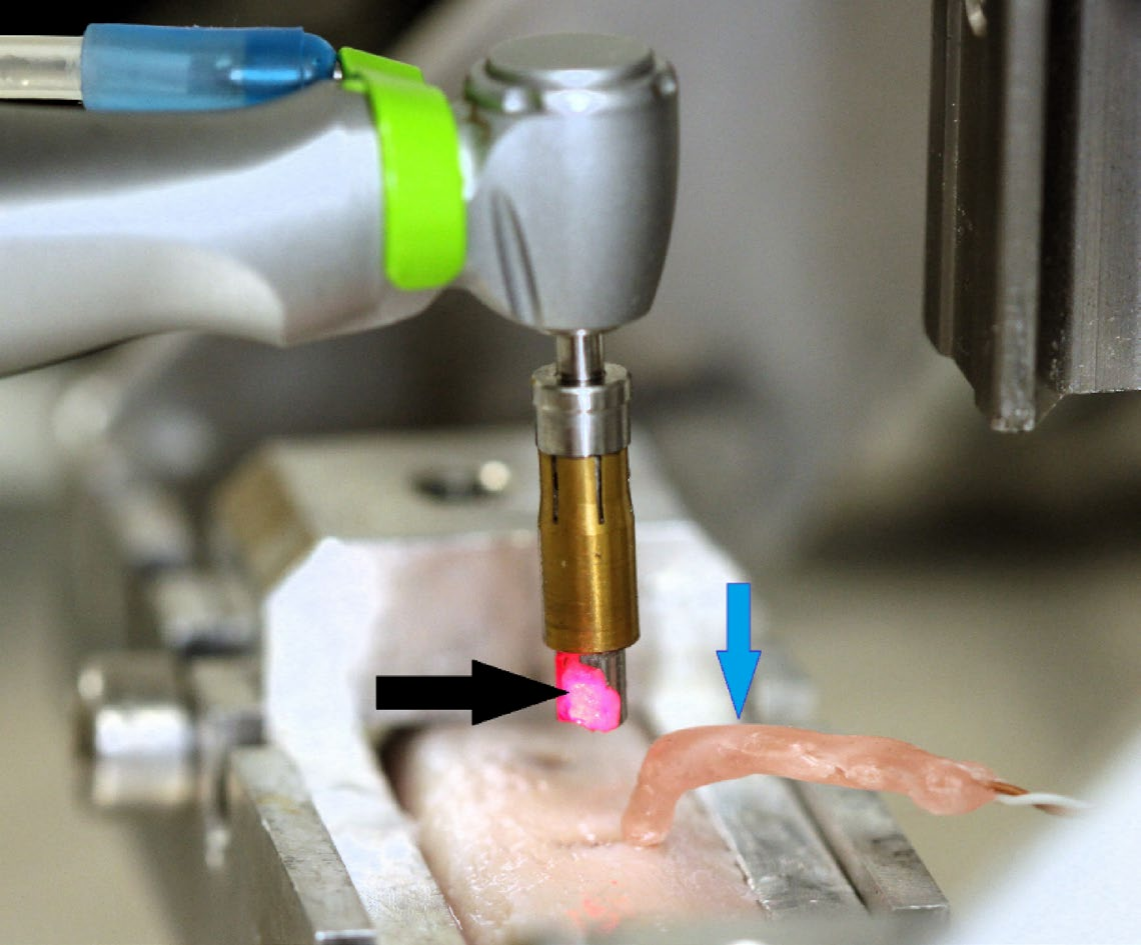
Intraosseous temperatures were continuously monitored using thermocouple probes (wax embedded sensors visible at blue arrow), while bone chip temperatures were measured by non-contact infrared device (measuring light visible at black arrow). Bone chip temperature was recorded within two seconds of completing the preparation.

All experiments were conducted in a dedicated, air-conditioned laboratory specifically designed for research purposes. Throughout the study, the room temperature was rigorously set and continuously maintained at 23 °C, ensuring a consistent and controlled experimental environment. Bone samples were immediately placed and continuously kept in physiologic saline in a regular fridge (5–7 °C) between drillings. Before the experiment, the specimens were transferred to another container with room temperature saline and were allowed to reach the temperature of the research room, which was monitored by a high-precision immersion/penetration probe (Pt100; TC Direct, Budapest, Hungary). Before the experiment, pig ribs were fastened tightly in a metal frame. Subsequently, a metal template was precisely positioned over the frame serving to determine and prepare temperature measuring holes. Temperature registering holes had a diameter of 0.5 mm, and a depth of 5 mm and they were located ~ 0.5 mm from the final periphery of the bone chips collecting preparations (Fig. 2). To disclose the influence of irrigation fluid to the probe’s pure metal parts, dental wax was used to perfectly seal the entrance of the temperature sensor cavity from any possible fluid penetration (Figs. 1 and 3). For continuous recording of intrabony temperature changes during the experiment, a Cu/CuNi thermocouple sensor (K-type, TC Direct, Budapest, Hungary) was inserted into the pre-drilled holes. This probe was connected to a data registration device (EL-EnviroPad-TC, Lascar Electronics Ltd., Salisbury, United Kingdom) via an insulated wire. This device recorded the temperature dynamics with a frequency of 1 measurement per second and a measurement accuracy of 0.1 °C.

Once drilling was finished, the drill was raised from the cavity by using the lever to elevate the tower’s axially moving part (Fig. 3). The temperature of the collected bone chips was measured using an infrared thermometer (TESTO 845, Testo Magyarország Kft., Budapest, Hungary). The temperature of the bone chips remaining in the drill was recorded at a distance of 7 cm. The device had a measurement resolution of 1 mm² and a stated accuracy of 0.1/1°C.

### Scanning electron miscroscopy

For scanning electron microscopy (SEM) analysis, the drills were subjected to ultrasonic cleaning (Realsonic, Senselektro, Budapest, Hungary) for five minutes using a 2% peracetic acid-based disinfectant solution (Sekusept Aktiv, Ecolab Europe GmbH, Zürich, Switzerland), followed by air drying. SEM imaging was conducted with a JSM-IT500HR microscope (JEOL, Tokyo, Japan) at magnifications ranging from 35x to 500x. Drill wear characteristics were assessed after both 10 and 30 preparation cycles.

### Statistical analysis

The statistical analyses were performed with SPSS v. 26.0 (IBM Corp., Armonk, NY, USA). The Kolmogorov-Smirnov test was applied to test the normality of the distribution of the data. To compare temperature data among different axial load and revolution speed groups, one way ANOVA was applied, followed by Bonferroni’s post hoc tests. When temperature productions of new and used drills were compared, the independent sample t-test was applied. To calculate the preparation time differences between the experimental groups, the non-parametric Kruskal-Wallis test (with pairwise comparisons) was used, since the distributions of the time data were found not normal. Preparation times of new and used drills were compared with Mann-Whitney U test. P values below 0.05 were considered significant.

## Results

### Donor bone temperatures

**Fig. 4:**
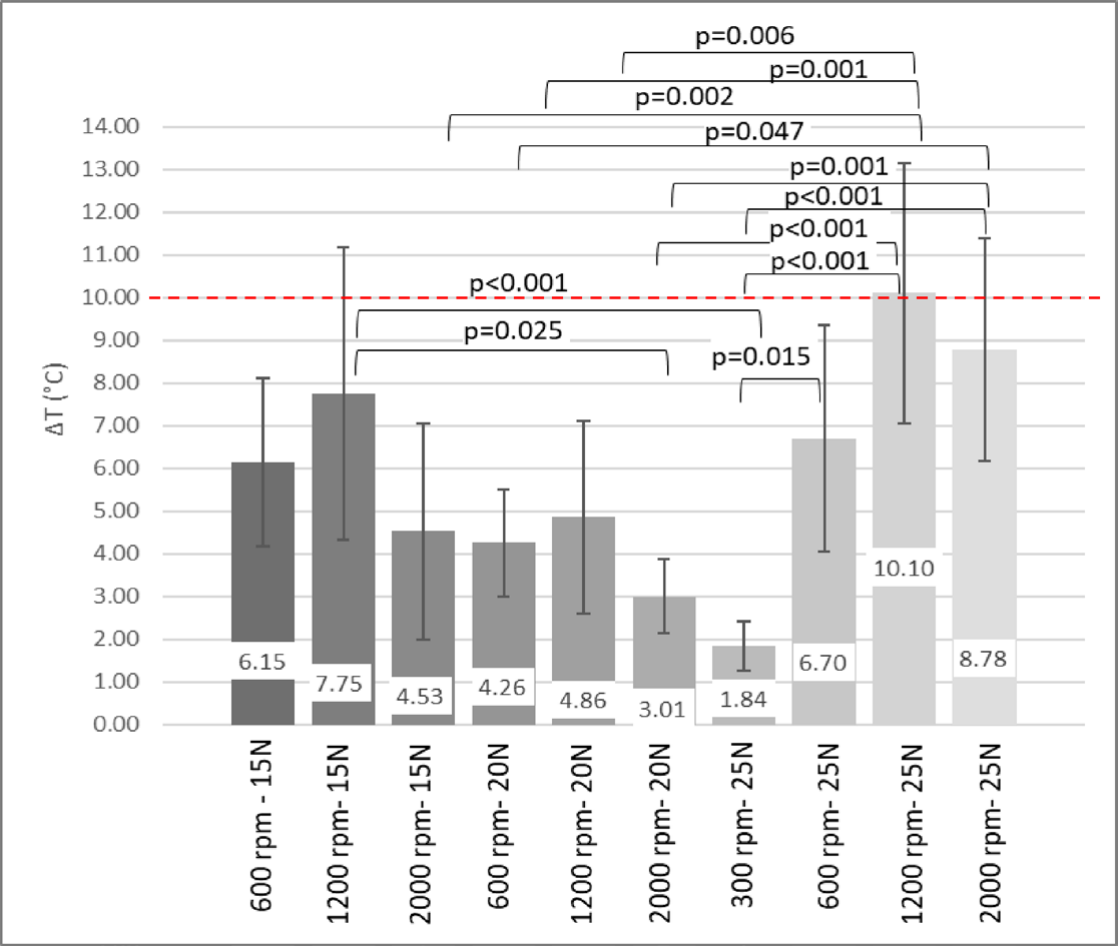
Temperature increase in donor bone samples by different drilling parameters. Temperatures of all the investigated revolutions and axial loads were compared simultaneously. ANOVA with Bonferroni post hoc test.

Figure 4 presents the temperature increases observed during drilling under various parameter settings. When applying a 25 N axial load, drilling at 1200 rpm produced the highest temperature increase (10.1 ± 3.04 °C; *p* = 0.021 and *p* < 0.001). In contrast, drilling at both 600 rpm and 2000 rpm resulted in lower temperature elevations, with no statistically significant difference between these two groups (*p* = 0.721). Upon application of a 20 N axial load, temperature increases were comparable across the 600, 1200, and 2000 rpm groups, with recorded values ranging from 3.01 °C to 4.86 °C. When a 25 N axial load was applied, drilling at 300 rpm produced the lowest mean temperature increase (1.84 ± 0.58 °C; *p* < 0.001).

At axial loads of 15 N and 20 N with a rotational speed of 300 rpm, the bone collecting drill was unable to penetrate fresh pig rib specimens. When the load was increased to 25 N at 300 rpm, some drills experienced fracture.

**Fig. 5:**
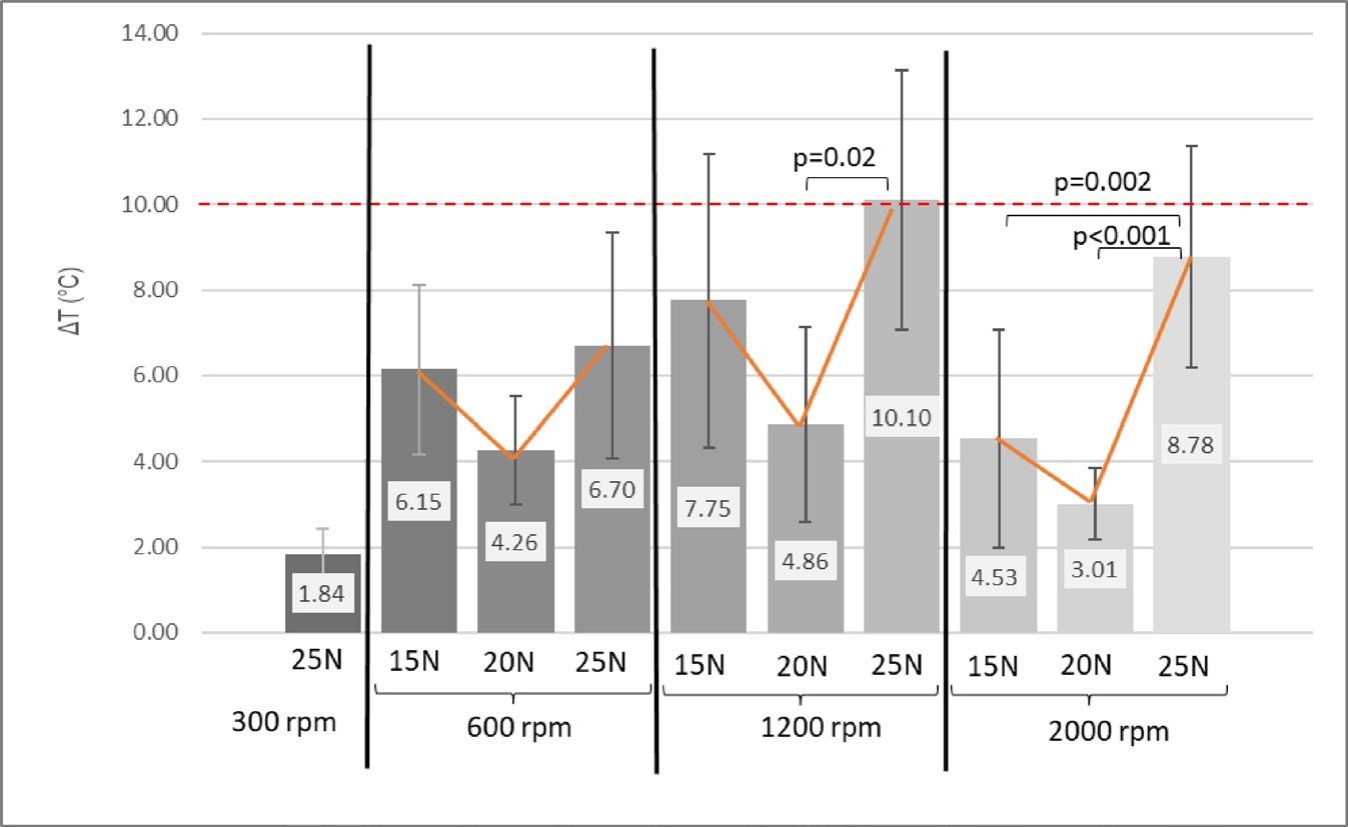
Temperature increases in donor bone samples by different drilling parameters. Temperatures were categorized and analyzed based on the corresponding identical revolutions. ANOVA with Bonferroni post hoc test.

With all drilling parameters considered, only the 1200 rpm group exceeded the 10 °C osteonecrosis threshold at 15 N axial load, whereas both 1200 and 2000 rpm groups did so at 25 N. When temperatures were organized according to drilling revolutions for improved visualization (see Fig. 5), it was observed that only drillings conducted at 300 and 600 rpm maintained temperatures below the 10 °C threshold. At higher speeds of 1200 rpm and 2000 rpm, certain axial loads led to temperature increases exceeding this limit.

### Bone fragment temperatures

**Fig. 6:**
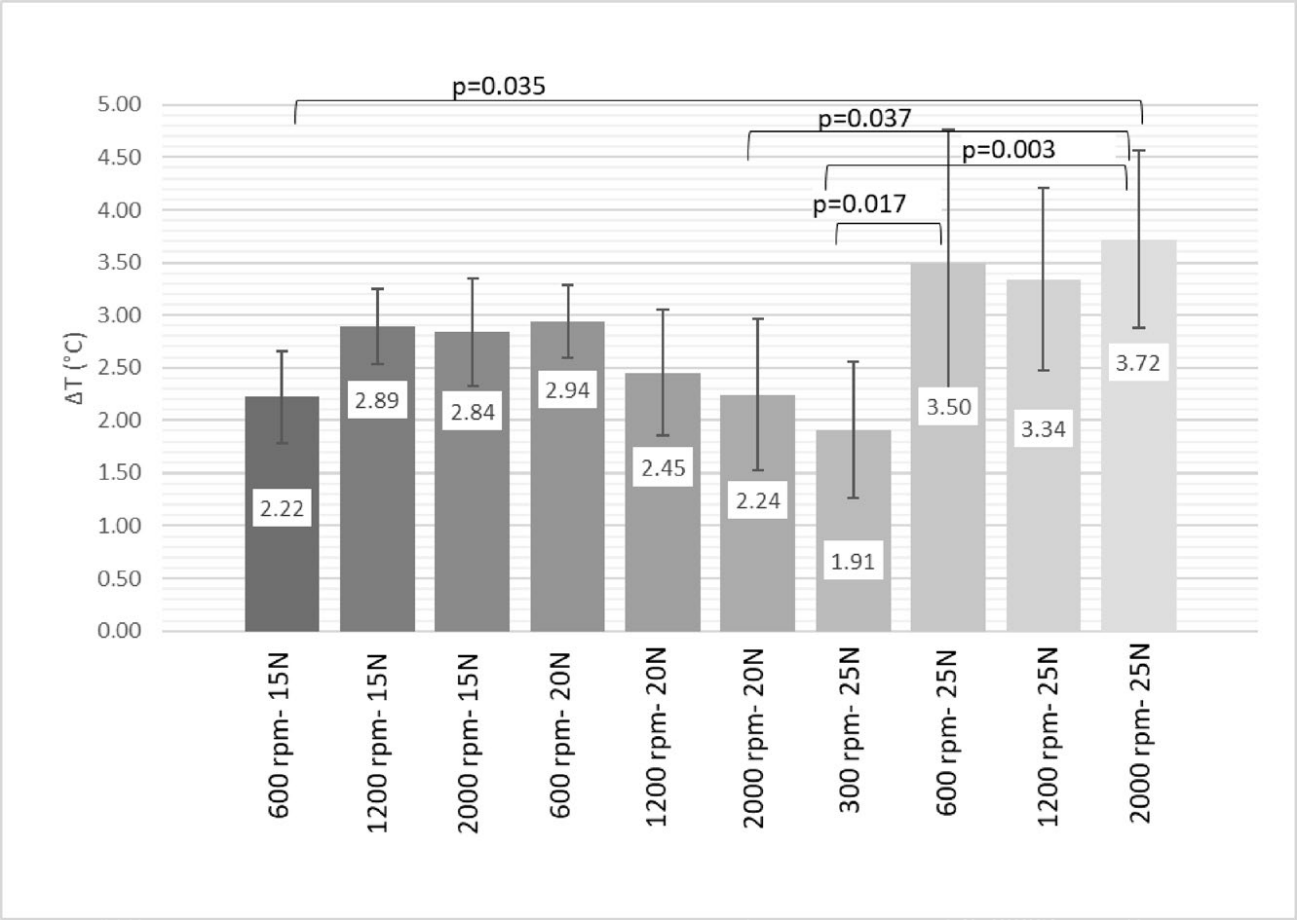
Temperature increases in collected bone chip samples by different drilling parameters. The temperatures at all investigated revolutions and axial loads were compared simultaneously. ANOVA with Bonferroni post hoc test.

Temperature increases in collected bone chips after preparation stayed below 5 °C (Fig. 6). In the 15 N group, drilling at 600 rpm produced the lowest temperature rise at 2.22 ± 0.44 °C (*p* = 0.013, *p* < 0.001). In the 20 N axial loading group, drilling speed did not affect results. In the 25 N group, 300 rpm produced significantly less heat than 600 rpm (*p* = 0.041) or 2000 rpm (*p* = 0.018).

### Preparation times

**Fig. 7:**
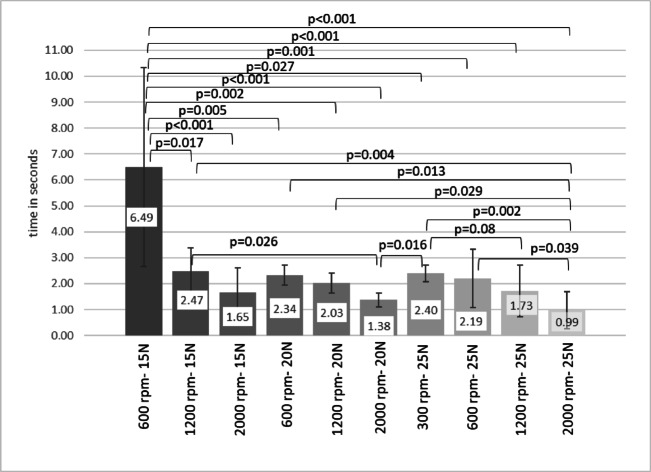
Preparation times of bone collecting drills by different setting parameters. All preparation times are clinically acceptable.

In the 15 N group, preparations at 600 rpm were slower than at 1200 rpm (*p* = 0.006) and 2000 rpm (*p* < 0.001). With a 20 N axial load, drilling at 2000 rpm was faster than at 600 rpm (*p* = 0.035) or 1200 rpm (*p* = 0.025). For a 25 N axial load, a statistically significant difference was identified only between drilling at 300 rpm (2.40 ± 0.31s) and 2000 rpm (0.99 ± 0.72s) (*p* < 0.001). When considering all drilling parameters together, the longest mean preparation time (6.49s) occurred at 15 N and 600 rpm, whereas the shortest times (25 N: 0.99s, 20 N: 1.38s, 15 N: 1.65s) were recorded with 2000 rpm drillings (Fig. 7).

### Drill wear

**Fig. 8:**
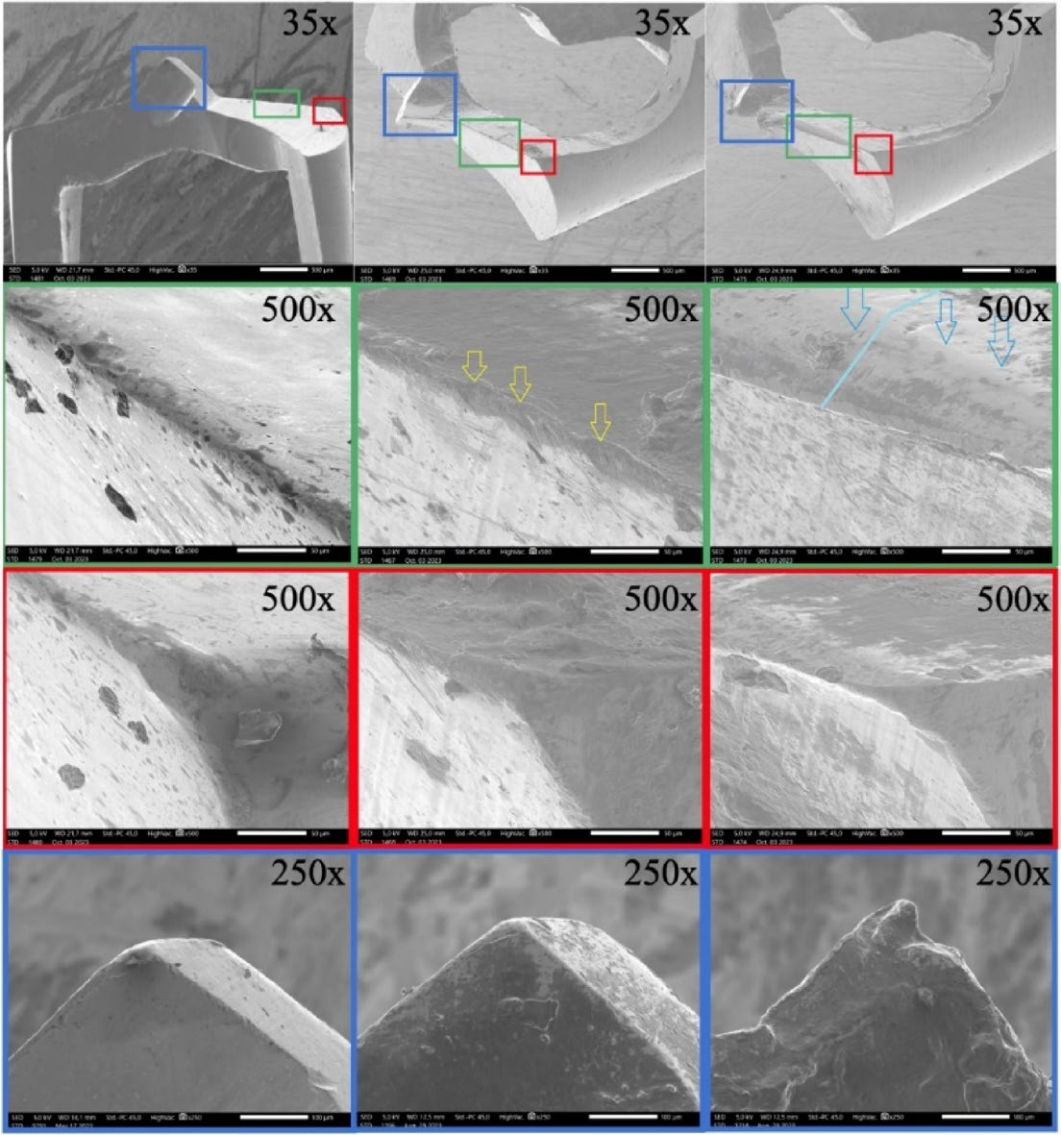
The Scanning Electron Microscopic images display new drills (left column), drills after ten uses (middle column), and drills used thirty times (right column). Green boxes indicate the cutting edge at 500x magnification, red boxes show the corner of the cutting edge at the cylinder wall (= body clearance) of the drill at 500x magnification, and blue boxes represent the drill apex (= chisel edge) at 250x magnification. Increased wear is evident across various sections of the drill from left to right. Minor deformation of the cutting edge was observed after ten uses (indicated by yellow arrows), whereas significant abrasion became evident following thirty uses (highlighted by the blue line and blue arrows). After 30 uses, significant deformation was observed at the apex, and the tip exhibited bifurcation. Drill fracture should be anticipated under these conditions.

**Fig. 9:**
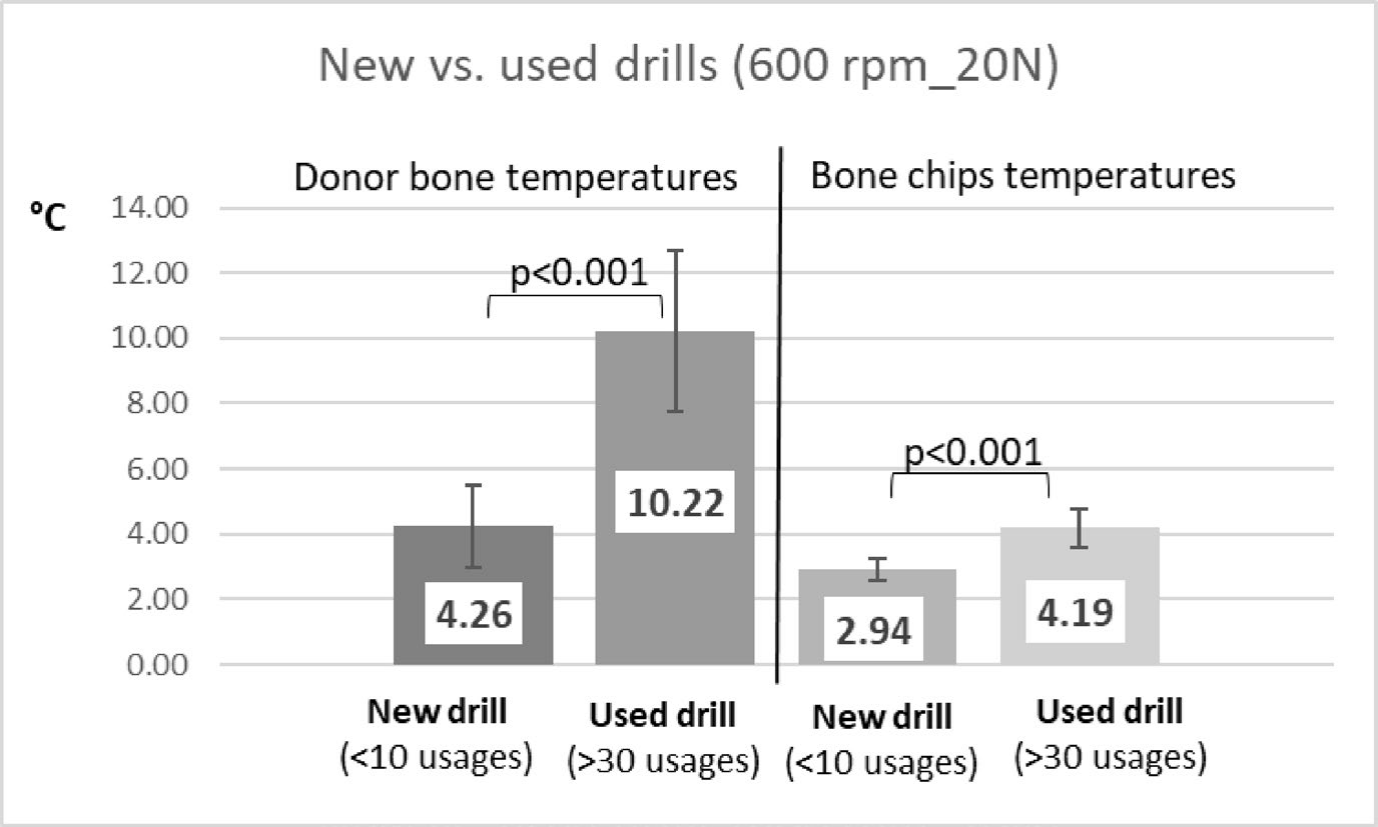
The diagram presents data on temperature elevations within the donor bone cavity and in collected bone fragments when employing either new drills or those subjected to 30 prior uses. While notable increases in temperature were observed, clinical significance pertains exclusively to the donor bone with respect to the potential risk of harmful thermal exposure.

SEM analysis revealed notable wear after 30 uses, with a clearly rounded and deformed cutting edge (see blue lines and arrows in Fig. 8). The drill tips showed considerable deformation, and many fractured at the tip—often forming a double tip separated by a fracture line—after around 20–30 uses. With respect to the impact of drill wear on donor bone temperatures, it was observed that worn drills generated significantly greater intraosseous heat compared to new drills (independent sample t-test, *p* < 0.001) (Fig. 9). Bone chip temperatures collected using worn drills were also significantly elevated (independent samples t-test, *p* < 0.001); however, the maximum temperature increases did not exceed 5 °C (Fig. 9).

Preparation times were notably longer when worn drills were utilized (4.39 ± 0.67s) compared to new drills (2.34 ± 1.17s), as determined by the Mann-Whitney U test (*p* = 0.003).

## Discussion

This study investigated the optimal parameters for autologous bone collecting drills using a porcine bone model. Considering the geometrical characteristics of the drill type examined, our objective was to determine the most suitable values for rotational speed and axial load with respect to drill wear, ensuring that heat generation remains below the theoretical threshold for harmful bone damage of 10 °C^25^.

The optimal drilling procedure for autologous bone harvesting is contingent on numerous factors, analogous to those that pertain to dental implant bed preparations. These include local bone characteristics, the various drill and drilling parameters, and the surgeon’s experience^10,12,26^. Several authors described a temperature increase in accordance with rotational speed increase^11,27–29^. Nevertheless, expedited preparations can result in reduced exposure to potentially harmful noxae. It is interesting to note that increasing only the rotational speed was not sufficient to elicit higher temperature values in the donor area in the present study. Moreover, research has indicated that elevated feed rates result in diminished temperature increments^27,29–31^. However, the drilling speed range (low vs. high-speed drilling in oral surgery) or drill shape (twist drills, round drills, trephines or special bone collecting drills) and design (flutes and helices, cutting edges etc.) may highly influence these correlations^32,33^. Higher rotational speeds generally increase the rate of friction between the drill and bone, leading to greater heat generation. As the drill rotates faster, the cutting edges interact with the bone more frequently per unit time, which increases the energy transferred as heat. However, at very low speeds, prolonged contact time can also allow heat to accumulate locally, especially if the feed rate is not adjusted accordingly. The relationship is not strictly linear, as optimal speeds may minimize heat by balancing cutting efficiency and frictional losses^9–12,14^. Increased axial force can enhance cutting efficiency by allowing the drill to penetrate more effectively, reducing the duration of drilling and, consequently, the total heat exposure. However, excessive axial force increases friction and may cause microcracks or mechanical trauma, which can also contribute to heat generation. There is an optimal range where the force is sufficient for efficient cutting but not so high as to cause excessive friction or damage^9–12,14^. According to our results, in case of bone collecting drills, there was no linear correlation between revolutions or axial load and temperature increases. Elevating the axial load from 15 to 20 N resulted in a decline in the average heat development, which remained within the safety zone in the majority of the examined groups. An exception was observed in the case of 300 revolutions per minute (rpm). However, when the axial load was increased from 20 N to 25 N, a clear increase in temperature was observed. In a clinical context, the applied axial load may be significantly influenced by surgeons’ behaviors^13^. Less effective, worn drills frequently evoke higher axial pressure from the surgeons to achieve equivalent preparation performance. This is achieved by simultaneously increasing bone thermal loading. Geometrical properties of the drills play an important role in heat development^32,33^. According to the most current knowledge available, a consensus has yet to be reached regarding the optimal design and characteristics of bone collecting drills. A review of the literature on twist drills reveals a divergence of opinion regarding the influence of the point angle of the drill bit on temperature development. While some researchers have indicated that the point angle is inconsequential^27,30^, others have identified a clear correlation between the extent of the point angle and the produced heat^28,34^. However, the point angle of the investigated bone collecting drill underwent significant modification during its usage due to wear. Approximately after 30 applications, the drill’s apex underwent substantial alterations, manifesting as notable wear (e.g., a rounded apex and chipping of the cutting edge). In some cases, complete vertical fractures developed at the tip. Studies on drilling forces indicate that majority of mechanical work is carried out by the two cutting edges and the chisel point of the drill bit, with minimal contribution from the drill bit margin^35^. Most of the mechanical energy during drilling is converted into heat, part of which is transferred to the bone^36,37^. Sui et al., determined that heat distribution around the drilled cavity is affected by drill bit geometry, drilling process parameters, and bone type^38^. Consequently, the maximum temperature increase is predicted to occur at the interface between the cavity wall’s surface and the drill bit. Furthermore, the presence of chips in the flutes necessitates increased effort, resulting in a substantial rise in temperature^38^. Furthermore, the migration of bone fragments is a critical component in the process of eliminating heat from the cavity. This is a function that bone collecting drills are incapable of performing. In addition, the contemporary drill is equipped with an outer metal sleeve, which has the potential to induce heat accumulation within the drill’s interior environment. This design also ensures the separation of irrigation fluid from the accumulated bone chips.

Accurately measuring temperature increases at the preparation interface remains a complex task. Many researchers prefer intraosseous temperature measurement using thermocouple probes, whereas others opt for the simpler non-contact thermal registration, primarily utilizing infrared technology. Thermal sensors affixed to the bone surface are also employed^22^. In a study conducted by the present authors, it was demonstrated that non-contact heat registration yielded significantly lower temperature readings compared to simultaneously placed intraosseous sensors^39^.

Maintaining appropriate cooling and irrigation during bone preparation is essential to prevent temperatures from exceeding the 47 °C threshold^12,25^. The literature offers varying perspectives and outcomes regarding factors such as internal versus external cooling methods, optimal fluid volume, and the temperature of the irrigant^20,40^. According to a systematic review, low-speed drilling (< 300 rpm) without irrigation can be employed for implant osteotomy preparations^41^. Nonetheless, in guided implantology, surgical guides are often cited as obstacles to effective irrigation at the drilling site. Tuce et al., demonstrated that internal coolant markedly reduces bone heating during guided implant osteotomy, even in the absence of a specific irrigation evacuation channel, provided the surgical template does not rest directly on the target surface^42^. Barrak et al. observed that cooled irrigation can adequately compensate when fluid contact with the drill surface is restricted by guide sleeves^20^. It should be noted that the irrigation used in this study was optimal in terms of amount (approximately 90 ml/min) and free of the disturbing factors that can be present in real clinical situations, such as the rear retromolar location and mouth opening limitations, excessive tongue or buccal soft tissues, or a partially covering mucoperiosteal flap^13^. In clinical scenario when irrigation is less or limited, higher temperatures can also be expected.

Repeated use or wear of drills contributes to increased surface roughness, which in turn elevates friction and results in higher operating temperatures. Despite these observations, comprehensive guidelines and robust evidence-based data remain insufficient. Scanaro et al., investigated the effects of drill wear on temperature elevation, excluding coolant and sterilization cycles, and observed a significant temperature rise after 30 uses—findings consistent with those of the present study^43^. They identified drill wear as a primary contributor to increased friction, attributed to enhanced surface roughness at the cutting edge. Chacon et al. examined heat generation across three implant systems, this time including sterilization cycles. Their results indicated that two drill types could be used safely up to 25 times, whereas the third type demonstrated temperature increases exceeding 10 °C, likely due to its structural design^32^. In our study, we found that worn drills resulted in significantly higher temperatures in both the donor site and the harvested bone compared to new drills; however, critical temperature elevations (> 10 °C) were observed only in the donor bone.

This study has certain limitations. As the research was performed ex vivo using porcine rib models, it does not directly reflect human cellular viability or actual physiological and pathological tissue responses to the thermal or postulated mechanical trauma observed. Since cortical thickness was homogenic in this study, human variations including thicker corticals in certain locations should be considered^44^. Additionally, temperatures were recorded at approximately 0.5 mm from the preparation cylinder walls, while higher temperature readings could be present, particularly in the apical regions of the donor bone cavities. Temperature measurements of bone chips using non-contact infrared thermography are subject to uncertainty due to the difficulty in precisely determining the emissivity of the chips and the very small cross-sectional area being measured. These factors may introduce measurement error, and results should be interpreted with this limitation in mind. SEM analysis was limited to qualitative and subjective assessment through visual inspection and could not provide quantitative measurements of wear. It is recommended that future research also examine different bone collecting drill designs produced by other manufacturers.

## Conclusions

All drilling parameters assessed in this study were appropriate for bone chip harvesting, as peak temperatures did not exceed 5 °C. Regarding temperature increases at the donor site, an axial load of 20 N (regardless of rotation speed) or drilling at 600 rpm (regardless of axial load) is recommended. Drills are not recommended for use more than 30 times, and regular visual inspection of drill wear—especially at the tip, looking for pre-fracture signs—should be conducted even before reaching 30 uses.

## Data Availability

Data supporting findings of this study are available from corresponding author upon reasonable request.
